# Adiponectin Signaling Modulates Fat Taste Responsiveness in Mice

**DOI:** 10.3390/nu16213704

**Published:** 2024-10-30

**Authors:** Fangjun Lin, Emeline Masterson, Timothy A. Gilbertson

**Affiliations:** 1 Burnett School of Biomedical Sciences, College of Medicine, University of Central Florida, Orlando, FL 32827, USA; linfj96@gmail.com (F.L.); emeline.masterson@ucf.edu (E.M.); 2 Department of Internal Medicine, College of Medicine, University of Central Florida, Orlando, FL 32827, USA

**Keywords:** adiponectin, fat taste, fatty acid, CD36, adipoR1, calcium imaging

## Abstract

Background/Objectives: Adiponectin, the most abundant peptide hormone secreted by adipocytes, is a well-known homeostatic factor regulating lipid metabolism and insulin sensitivity. It has been shown that the adiponectin receptor agonist AdipoRon selectively enhances cellular responses to fatty acids in human taste cells, and adiponectin selectively increases taste behavioral responses to intralipid in mice. However, the molecular mechanism underlying the physiological effects of adiponectin on fat taste in mice remains unclear. Conclusions: Here we define AdipoR1 as the mediator responsible for the enhancement role of adiponectin/AdipoRon on fatty acid-induced responses in mouse taste bud cells. Methods and Results: Calcium imaging data demonstrate that AdipoRon enhances linoleic acid-induced calcium responses in a dose-dependent fashion in mouse taste cells isolated from circumvallate and fungiform papillae. Similar to human taste cells, the enhancement role of AdipoRon on fatty acid-induced responses was impaired by co-administration of an AMPK inhibitor (Compound C) or a CD36 inhibitor (SSO). Utilizing *Adipor1*-deficient animals, we determined that the enhancement role of AdipoRon/adiponectin is dependent on AdipoR1, since AdipoRon/adiponectin failed to increase fatty acid-induced calcium responses in taste bud cells isolated from these mice. Brief-access taste tests were performed to determine whether AdipoRon’s enhancement role was correlated with any differences in taste behavioral responses to fat. Although AdipoRon enhances the cellular responses of taste bud cells to fatty acids, it does not appear to alter fat taste behavior in mice. However, fat-naïve *Adipor1*^−/−^ animals were indifferent to increasing concentrations of intralipid, suggesting that adiponectin signaling may have profound effects on the ability of mice to detect fatty acids in the absence of previous exposure to fatty acids and fat-containing diets.

## 1. Introduction

Adiponectin, a hormone derived from adipose tissue, acts as a messenger between fat tissue and other organs and plays essential roles in regulating energy homeostasis, obesity, diabetes, cardiac function, and inflammation. For example, adiponectin inhibits gluconeogenesis in the liver and enhances fatty acid oxidation in skeletal muscle, contributing to beneficial metabolic effects in energy homeostasis [1]. Adiponectin can improve endothelial dysfunction caused by elevated free fatty acids [2,3]. Adiponectin protects against obesity-related glomerulopathy and diabetic nephropathy [4,5]. Moreover, adiponectin has been found to enhance 5′ adenosine monophosphate-activated protein kinase (AMPK) activity in the hypothalamus, stimulating food intake [6]. Adiponectin can also be produced by salivary gland epithelial cells [7], and salivary adiponectin has been identified in several studies [8,9,10]. In addition, high expression levels of adiponectin receptors were observed in mouse taste bud cells [11] and immortalized human fungiform (HuFF) taste cells [12]. Thus, these studies suggest a potential role for adiponectin signaling in taste perception.

There is growing evidence that taste function can be modulated by hormones that act on their receptors in the peripheral gustatory system. Of the many hormones that influence taste perception, several appear to modulate cellular, neural, or behavioral responses to fatty acids, such as leptin, peptide YY (PYY), glucagon-like peptide-1 (GLP-1), ghrelin, cannabinoids, and adiponectin. For example, leptin suppresses cellular and neural responses to fatty acids [13,14]. Behavioral responses to lipid emulsions were attenuated in PYY knockout mice and rescued by the targeted augmentation of salivary PYY via viral vector therapy [15]. Administration of the GLP-1 receptor agonist exendin-4 decreased taste responses to intralipid [16], and *Glp1r* knockout mice lost the ability to detect low concentrations of lipids [17]. Reduced taste sensitivity to intralipid was observed in ghrelin and ghrelin O-acyltransferase (GOAT) knockout mice [18], and ghrelin receptor (growth hormone secretagogue receptor, *Ghsr*) knockout females but not males showed a reduction in taste responsiveness to linoleic acid [19]. Cannabinoid 1 receptor (*Cb1r*)-deficient mice displayed a low preference for fatty solutions compared to wild-type mice [20]. Moreover, salivary gland-specific adiponectin rescue significantly increased behavioral taste responses to intralipid in adiponectin knockout mice [11]. We recently reported that adiponectin selectively enhances fatty acid-induced calcium responses in immortalized human fungiform taste cells by increasing the surface expression of CD36 [12]. However, the physiological connection between adiponectin signaling and fat taste sensitivity in the native taste system, if any, is largely unexplored.

Adiponectin acts mainly on three receptors, including AdipoR1, AdipoR2, and T-cadherin. These receptors are widely distributed throughout the body, with AdipoR1 most abundantly expressed in skeletal muscle and AdipoR2 predominantly expressed in the liver [21]. Immunohistochemical staining showed that AdipoR1 and T-cadherin immunolocalize to mouse circumvallate taste buds, while AdipoR2 immunolocalizes to the surrounding tissues, but not the taste buds [11]. AdipoR1 and AdipoR2 mediate different pathways of adiponectin downstream signaling and show opposing effects on energy metabolism in mice [22]. AdipoR1 appears more tightly associated with activating the AMPK pathway, whereas AdipoR2 is more closely involved in activating the peroxisome proliferator-activated receptor (PPAR)α pathway [23].

Additionally, our previous study showed that the EC_50_ of AdipoRon’s enhancement of linoleic acid (LA)-induced calcium response is close to the reported Kd of AdipoRon binding to AdipoR1 and that the effect of AdipoRon on LA-induced calcium responses in HuFF cells requires the activation of AMPK [12]. We hypothesized that AdipoR1 may be responsible for the role of adiponectin in fat taste responsiveness. To explore this hypothesis, we first aimed to determine if AdipoRon/adiponectin could enhance fatty acid-stimulated calcium responses in mouse taste bud cells and whether AMPK and CD36 are required for this enhancement role. Next, we examined the contribution of AdipoR1 using taste bud cells isolated from *Adipor1* knockout mice. Following the functional studies in these cell-based assays, we also attempted to investigate the role of AdipoRon in regulating fat taste behavior in mice.

## 2. Materials and Methods

### 2.1. Animals

The mouse strain B6.129P2-Adipor1tm1Dgen/Mmnc (RRID: MMRRC_011599-UNC) was donated to the NIH-sponsored Mutant Mouse Resource and Research Center (MMRRC) by Deltagen. Heterozygous *Adipor1*^+/−^ mice were ordered from the MMRRC facility at the University of North Carolina. Adult C57BL/6J mice were obtained from Jackson laboratories or the Lake Nona animal facility at the University of Central Florida. Both male and female adult (2–6 months) mice were used to perform the calcium imaging experiments and the brief-access taste testing, and they were housed under standard laboratory conditions (12 h/12 h day/night cycle) with water and standard chow available ad libitum unless otherwise specified. The Institutional Animal Care and Use Committee of the University of Central Florida approved all animal procedures.

### 2.2. Genotype and Phenotype of the Adipor1-Deficient Mice

At about two weeks of age, newborn mice were ear-punched. Genomic DNA was extracted from ear punch samples with DirectPCR Lysis Reagent (Ear) and Proteinase K (Viagen Biotech, Los Angeles, CA, USA). Polymerase chain reaction (PCR) was performed with primers (sequences 5′ to 3′) as follows: NIH62-GS1, TCCACTGTGTCAGCTTCTCTGTTAC, NIH62-GS2, AGGCAGGGTAAGCTGATTAGCTATG, and NIH62-neo, GGGTGGGATTAGATAAATGCCTGCTCT (MMRRC Center Protocol 11599, www.med.unc.edu/mmrrc (accessed on 4 November 2022)). The PCR cocktail contained 1 μL crude ear lysates, 1 μL 20 μM primer mixture, 6 μL MyTaq™ Red Mix (Meridian Bioscience, Memphis, TN, USA), and 5 μL water. The PCR reaction was then carried out according to genotyping protocol 11,599 with initial heating to 94 °C for 5 min, then 36 cycles of 94 °C for 45 s, 60 °C for 45 s, and 72 °C for 90 s, with a final extension at 72 °C for 7 min. After electrophoretic separation on 2% agarose gels, the expected sizes of bands (254 bp for wild type and 433 bp for mutant) were visualized with a ChemiDoc MP Imaging System (Bio-Rad, Hercules, CA, USA).

Body weights were recorded at about six weeks of age, and total body composition (fat mass, lean mass, and free body fluid) was determined using a Bruker time domain-nuclear magnetic resonance (TD-NMR, minispec LF50) live mice body composition analyzer (Bruker, Billerica, MA, USA). The homozygous *Adipor1* knockout (−/−) mice appeared normal at birth and developed and bred normally. Both male and female *Adipor1*^−/−^ mice were fertile. At about six weeks of age, the mean body weight of eight *Adipor1*^+/+^ male mice was 23.17 g, and that of eight *Adipor1*^+/+^ female mice was 18.42 g, while that of seven *Adipor1*^−/−^ male mice was 22.07 g and that of nine *Adipor1*^−/−^ female mice was 17.53 g (Figure S1A). The differences in the total body composition between *Adipor1^+^*^/*+*^ and *Adipor1*^−/−^ mice in males and females are shown in Figure S1B–D.

### 2.3. Chemicals and Solutions

Standard Tyrode’s solution contained (in mM) 140 NaCl, 5 KCl, 1 CaCl_2_, 1 MgCl_2_, 10 HEPES, 10 glucose, and 10 Na pyruvate; the pH was adjusted to 7.40 with NaOH; 300–320 mOsm. Calcium- and magnesium-free Tyrode’s solution contained (in mM): 140 NaCl, 5 KCl, 2 BAPTA, 10 HEPES, 10 glucose, and 10 Na pyruvate; the pH was adjusted to 7.40 with NaOH; 300–320 mOsm. Linoleic acid (LA) was purchased from Sigma (St. Louis, MO, USA), prepared as a stock solution (25 mg/mL) in 100% ethanol, and then stored under nitrogen at −20 °C. LA working solution (30 μM) was made from the stock solution immediately before use and only used on that day. Intralipid 20% IV fat emulsion was purchased from Patterson Veterinary (Loveland, CO, USA). The isolation enzyme cocktail components––collagenase A (0.5 mg/mL), dispase II (2 mg/mL), and trypsin inhibitor (1 mg/mL)––were ordered from Sigma. Stock solutions of AdipoRon (MedChem Express, Monmouth Junction, NJ, USA), sulfosuccinimidyl oleate (SSO; Cayman Chemical, Ann Arbor, MI, USA), and dorsomorphin (Compound C; ApexBio, Houston, TX, USA) were made in DMSO and diluted the day of the experiment to a designated concentration. R&D Systems recombinant mouse adiponectin protein was ordered from Fisher Scientific (Hampton, NH, USA).

### 2.4. Taste Cell Isolation

Individual taste bud cells were isolated from mouse circumvallate and fungiform papillae following procedures used in previous reports [24,25]. Briefly, control animals were sacrificed by exposure to CO_2_ followed by cervical dislocation. Tongues were removed and immediately placed in Tyrode’s solution. The anterior portion of the tongue containing the fungiform papillae and the area surrounding the circumvallate papillae were then injected with an enzyme cocktail in Tyrode’s solution. The injected tongue was incubated in Tyrode’s solution and bubbled with O_2_ for approximately 40 min. The lingual epithelium was separated from the underlying muscle layer and pinned flat in Tyrode’s solution in a Sylgard-coated petri dish. Next, the epithelium was incubated in calcium- and magnesium-free Tyrode’s solution for 5–7 min (males) and 3–5 min (females), washed with standard Tyrode’s solution, and subsequently incubated in the same enzyme cocktail described above for another 3–5 min (males) and 1–3 min (females). Taste bud cells were isolated by gentle suction using a fire-polished glass pipette under a dissection microscope and placed on coverslips coated with Corning Cell-Tak Cell and Tissue Adhesive (Corning, PA, USA) for calcium imaging.

### 2.5. Calcium Imaging

Intracellular calcium imaging was carried out on isolated taste bud cells loaded with 4 µM of the ratiometric calcium indicator Fura-2-acetoxymethyl ester (Fura-2 AM, Invitrogen, Waltham, MA, USA) in Tyrode’s with 0.05% pluronic acid F-127 (Invitrogen) for about 1 h at room temperature in the dark. The coverslips with isolated taste bud cells ready for imaging were placed into a perfusion chamber (RC-25F, Warner Instruments, Holliston, MA, USA). Taste stimuli solutions (30 μM LA with or without other compounds) were applied by a bath perfusion system at a flow rate of 4 mL/min for 3 min, followed by 1 min of 0.1% fatty acid-free BSA solution, and then regular Tyrode’s for about 2 min to remove the LA until the calcium signal returned to near baseline level. Taste bud cells were illuminated with a Lambda DG-5 illumination system (Sutter Instruments, Novato, CA, USA), and imaging was performed using an acA720 camera (Basler, Ahrensburg, Germany) through a 40× oil immersion objective lens on an Olympus CKX53 inverted microscope (Olympus, Tokyo, Japan). Taste bud cells loaded with Fura-2 AM were excited at 340 nm and 380 nm of light, and emission was recorded at 510 nm. Images were captured at a rate of 20 per min, and the ratio of fluorescence (340 nm/380 nm) was used to measure the changes in intracellular calcium levels on taste bud cells using InCyt Im2™ imaging software (Version 6.00, Cincinnati, OH, USA). Experiment 1: To observe the effect of AdipoRon on fatty acid-induced calcium responses in isolated taste bud cells from wild-type mice, LA (30 μM) and mixtures of LA (30 μM) with a series of AdipoRon concentrations (0.1, 1, 5, and 10 μM) were used. Experiment 2: LA (30 μM), a mixture of LA (30 μM) with AdipoRon (5 μM), and a mixture of LA (30 μM), AdipoRon (5 μM), and Compound C (an AMPK inhibitor, 10 μM) in random order were perfused over isolated wild-type mouse taste bud cells to link the activation of AMPK to the enhancement role of AdipoRon on fatty acid-induced calcium responses. Experiment 3: To investigate the involvement of CD36 in the effect of AdipoRon/adiponectin on fatty acid-induced responses, LA (30 μM) and mixtures of LA (30 μM) with AdipoRon (5 μM) or adiponectin (10 ng/mL) were perfused in random order over isolated wild-type mouse taste bud cells pretreated with SSO (an irreversible inhibitor of CD36, 100 µM, 30 min). Experiment 4: To determine whether the effect of AdipoRon/adiponectin on fatty acid-induced calcium responses is mediated by AdipoR1, LA (30 μM) and mixtures of LA (30 μM) with AdipoRon (5 μM) or adiponectin (10 ng/mL) were perfused in random order over taste bud cells isolated from *Adipor1*^−/−^ mice and *Adipor1*^+/+^ controls.

### 2.6. Brief-Access Taste Testing

Four groups of animals (*Adipor1*^+/+^ males, *Adipor1*^+/+^ females, *Adipor1*^−/−^ males, and *Adipor1*^−/−^ females; *n* = 8 for each group) were used for the brief-access testing. Mice were housed individually in standard cages and habituated to their environment for several days before testing began. Purified water was used to prepare test solutions. Brief-access testing was administered within an MS-160 Davis Rig gustatory behavioral apparatus (Med Associates, St. Albans, VT, USA). Water was restricted for about 21 h before each training and testing day (30 min test in Davis Rig, 1 h rest period, then free access to water for 1.5 h). Animals were trained by two steps: (1) one presentation of purified water for 15 min and a 15 min time limit to the first lick on the first day; (2) on days 2–3, training and testing on a series of sucrose solutions (25, 50, 100, and 200 mM) consisted of thirty 5 s presentations with 7.5 s intertrial intervals. Following the training phase, mice were subjected to five days of testing with intralipid solutions (0.5, 1, 5, 10, and 20%). The test sessions were 30 min, during which animals could initiate as many trials as possible. AdipoRon (10 μM) was added on testing days 3 and 4 to investigate its role in taste behavioral responses to intralipid. During training and testing, a fan was placed to direct airflow away from the stimulus presentation area to reduce the olfactory cues associated with the tastants.

### 2.7. Statistical Analysis

Calcium imaging data analyses were based on the amplitude of the intracellular calcium concentration and analyzed in Origin 9.6 (Version 9.6.0.172, OriginLab, Northampton, MA, USA). Due to the water deprivation, animals licked water or tastants profusely at the beginning of the brief-access taste testing. Once they had quenched their initial thirst, the number of water licks dropped dramatically compared to the preferred stimuli. To better represent the fundamental differences in taste behavioral responses between water and tastant, trials were included when the number of water licks began to drop dramatically. The tastant/water lick ratio was calculated by dividing the mean number of licks per trial for each concentration by the mean number of water licks per trial for that animal. Statistical analysis was performed using GraphPad Prism 10 (Version 10.2.1 (395), GraphPad Software, Boston, MA, USA). Unpaired Student’s *t*-test, one-way ANOVA, and two-way ANOVA were used as appropriate for each experiment. The level of significance was set at α = 0.05 for all experiments. All data are presented as mean ± SEM. For most bar graphs, individual values were not plotted due to the large number of points and conditions that made the graphs virtually uninterpretable visually.

## 3. Results

### 3.1. AdipoRon Enhances Cellular Responses to Fatty Acids in Mouse Taste Cells

**Figure 1:**
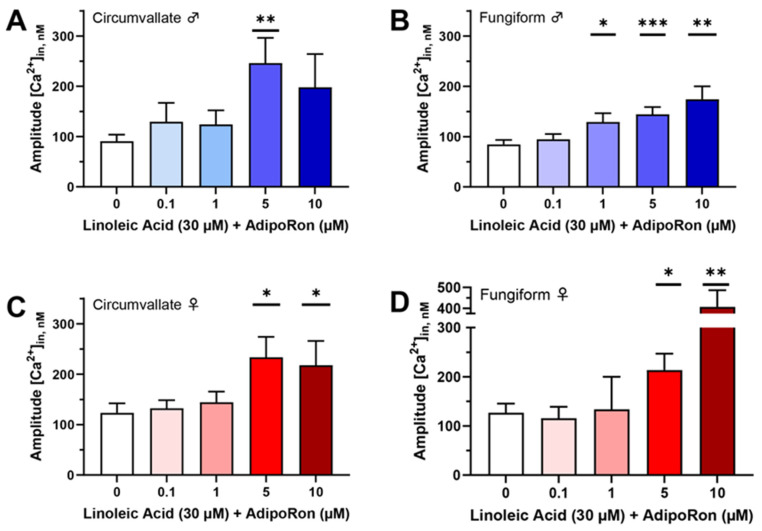
Mean amplitude of calcium responses to 30 µM linoleic acid (LA) with or without different concentrations of AdipoRon in mouse taste bud cells. (**A**) The LA-induced calcium response was significantly increased by 5 µM AdipoRon in circumvallate taste bud cells from male mice (t(78.53) = 2.995, *p* = 0.0037); (**B**) the LA-induced calcium response was significantly enhanced by 1 µM (t(206.9) = 2.253, *p* = 0.0253), 5 µM (t(331.1) = 3.49, *p* = 0.0005), and 10 µM (t(77.13) = 3.289, *p* = 0.0015) AdipoRon in fungiform taste bud cells from male mice; (**C**) the LA-induced calcium response was significantly increased by 5 µM (t(109.2) = 2.468, *p* = 0.0151) and 10 µM (t(126) = 2.114, *p* = 0.0365) AdipoRon in circumvallate taste bud cells from female mice; (**D**) the LA-induced calcium response was significantly enhanced by 5 µM (t(152.7) = 2.27, *p* = 0.0246) and 10 µM (t(50.06) = 3.409, *p* = 0.0013) AdipoRon in fungiform taste bud cells from female mice. Data are presented as mean ± SEM. Unpaired, two-tailed Student’s *t*-test (with Welch’s correction if the standard deviations of the populations are not equal) was used to determine statistical significance for the LA response compared with the treatments with AdipoRon. Asterisks correspond to significant differences between the AdipoRon-treated group and LA alone control, * *p* < 0.05, ** *p* < 0.01, *** *p* < 0.001.

We recently reported that the adiponectin receptor agonist AdipoRon enhances fatty acid-induced calcium responses in a human fungiform (HuFF) cell line (T0029; Applied Biological Materials, Richmond, BC, Canada). To determine if there is a similar enhancement role of AdipoRon on calcium responses to fatty acids in isolated native mouse taste bud cells, we examined intracellular calcium levels in isolated circumvallate and fungiform taste bud cells stimulated with 30 μM linoleic acid (LA) and 30 μM LA and different concentrations of AdipoRon (0.1, 1, 5, and 10 μM). Similar to our results in HuFF cells, AdipoRon enhanced the 30 µM LA-evoked calcium responses in a dose-dependent manner, with little to no effect seen below 1 μM and an obviously noticeable enhancement above 5 μM in isolated fungiform and circumvallate taste bud cells from both male and female mice (Figure 1A–D, *n* = 30−197 cells per point from at least three animals for each group).

### 3.2. The Effect of AdipoRon on LA-Induced Responses Is Mediated by AMPK Activation in Mouse Taste Cells

**Figure 2:**
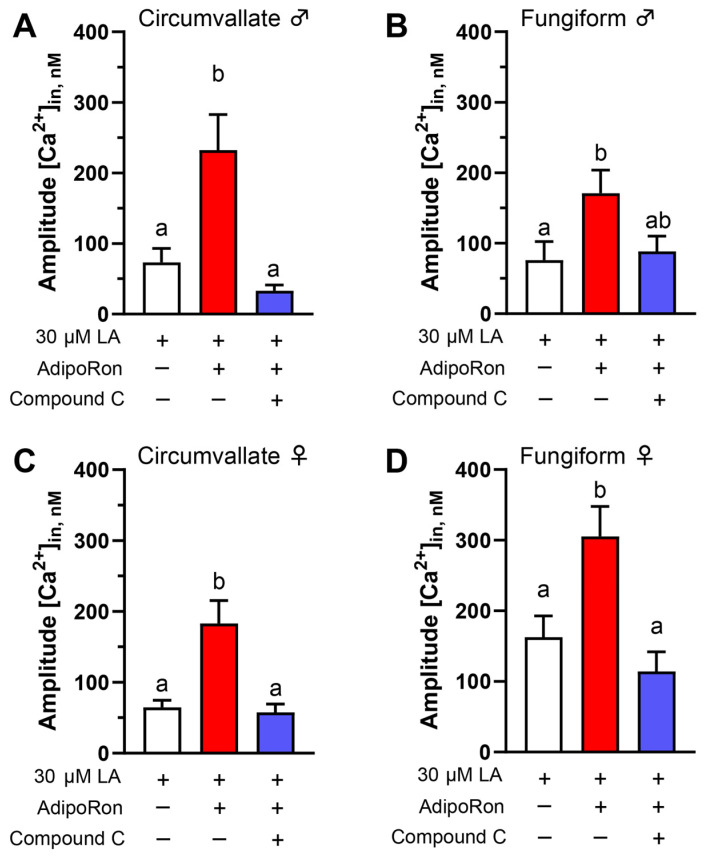
An AMPK inhibitor (Compound C) eliminates AdipoRon’s ability to enhance LA-induced calcium responses in mouse taste bud cells. The enhancement of taste cell responses to 30 μM LA caused by application of 5 μM AdipoRon was effectively eliminated by the addition of 10 μM Compound C in mouse taste bud cells from (**A**) male circumvallate (F*(2, 51.63) = 10.97, *p* = 0.0001; W(2, 58.43) = 8.772, *p* = 0.0005), (**B**) male fungiform (F(2, 105) = 3.562, *p* = 0.0319), (**C**) female circumvallate (F*(2, 48.23) = 11.35, *p* < 0.0001; W(2, 60.16) = 6.545, *p* = 0.0027), and (**D**) female fungiform (F*(2, 137.6) = 8.508, *p* = 0.0003; W(2, 103.3) = 7.086, *p* = 0.0013) taste cells. Data are presented as mean ± SEM. One-way ordinary ANOVA with Tukey’s test for multiple comparisons was used to determine statistical significance with equal standard deviations. Brown–Forsythe and Welch ANOVA tests with Dunnett’s T3 multiple comparisons test were introduced when standard deviations significantly differed. The letters above the bars indicate statistically significant groups within each cell type.

AdipoRon increases the phosphorylation state of AMPKα (Thr172) in HuFF cells, and AdipoRon’s ability to enhance LA-induced response is associated with activation of AMPK [12]. Therefore, we used a pharmacological approach to test whether activation of AMPK mediates the enhancement role of AdipoRon on fatty acid-induced calcium responses in isolated mouse taste bud cells in a similar fashion. The enhancement of taste bud cell responses to LA (30 μM) caused by the application of AdipoRon (5 μM) was effectively eliminated by the addition of AMPK inhibitor Compound C (10 μM). This acute response was found in circumvallate and fungiform taste papillae cells isolated from male and female mice (Figure 2A–D).

### 3.3. Adiponectin/AdipoRon Acts on CD36 to Increase LA-Induced Responses in Mouse Taste Cells

**Figure 3:**
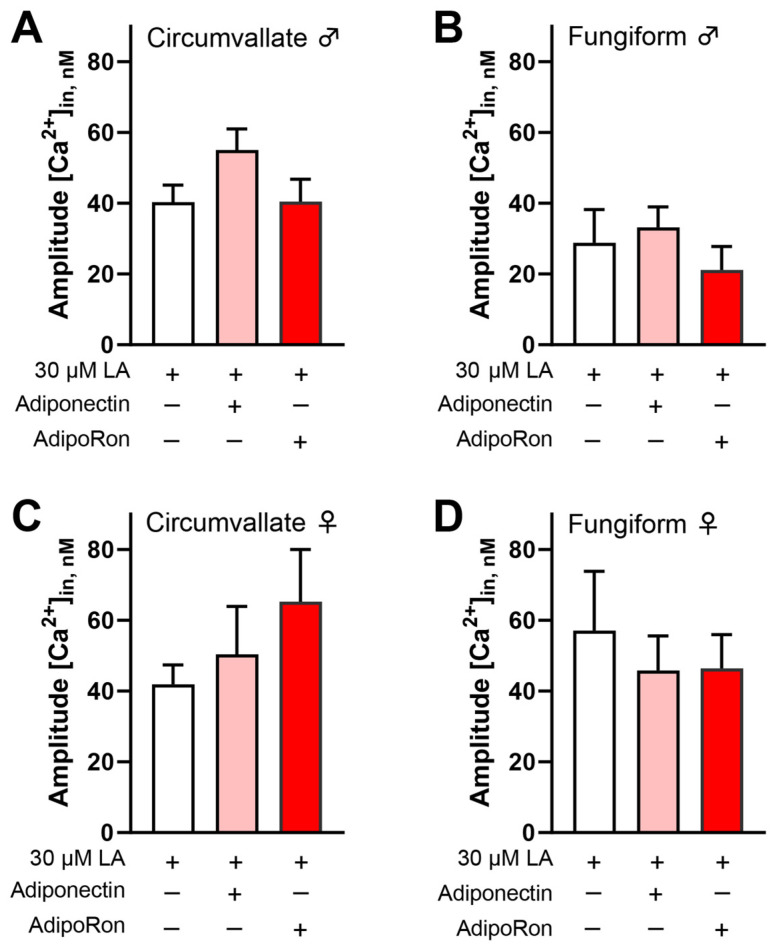
Blocking CD36 with sulfosuccinimidyl oleate (SSO) attenuated the enhancement role of AdipoRon/adiponectin on LA-induced calcium responses in mouse taste bud cells. No significant differences in calcium responses were observed in 100 µM SSO-pretreated taste bud cells (isolated from (**A**) male circumvallate; (**B**) male fungiform; (**C**) female circumvallate; and (**D**) female fungiform papillae) stimulated with 30 μM LA with or without 5 μM AdipoRon and 10 ng/mL adiponectin (*p* > 0.05 for all tests; *n* = 44−71 cells from at least three animals for each group). Data are presented as mean ± SEM. Unpaired, two-tailed Student’s *t*-test (with Welch’s correction if the populations’ standard deviations are not equal) was used to determine statistical significance between the adiponectin- or AdipoRon-treated group and the LA-alone control.

In HuFF cells, we found that AdipoRon’s effect on enhancing fatty acid-induced calcium responses is mediated by CD36 signaling but not GPR120 signaling [12]. Here, we used a pharmacological approach to investigate the involvement of CD36 in the effect of AdipoRon/adiponectin on fatty acid-induced responses. Mouse taste bud cells were pre-incubated with SSO, an irreversible inhibitor of CD36 (100 µM), for 30 min. Following incubation, cells were perfused LA (30 μM) and mixtures of LA (30 μM) with AdipoRon (5 μM) or adiponectin (10 ng/mL) in random order. Figure 3 shows that no significant differences in calcium responses were observed in SSO-pretreated taste bud cells stimulated by LA with or without AdipoRon or adiponectin (Figure 3A–D).

### 3.4. Adiponectin/AdipoRon Enhancement of LA-Induced Responses Is Dependent upon AdipoR1

**Figure 4:**
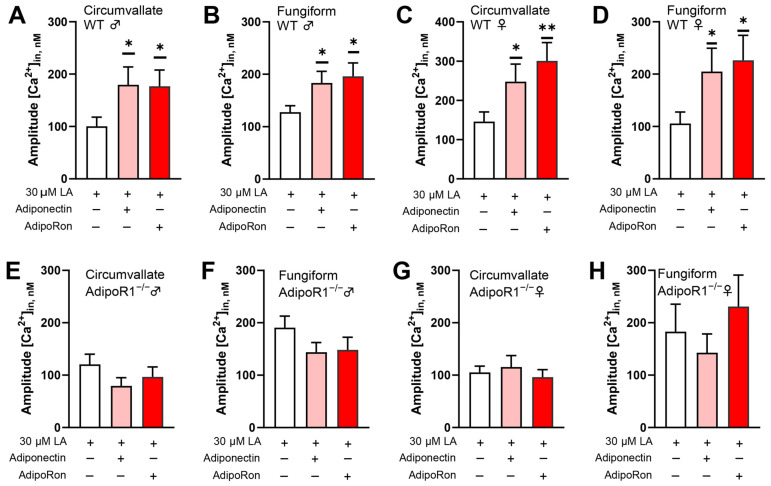
Adiponectin/AdipoRon enhancement of fatty acid responses is dependent upon AdipoR1. (**A**) Application of 10 ng/mL adiponectin (t(76.57) = 2.069, *p* = 0.0419) and 5 μM AdipoRon (t(81.07) = 2.153, *p* = 0.0343) significantly increased the 30 μM LA-induced calcium responses in mouse taste bud cells from circumvallate papillae from *Adipor1*^+/+^ males; (**B**) application of adiponectin (t(110.6) = 2.173, *p* = 0.0319) and AdipoRon (t(102.5) = 2.408, *p* = 0.0178) was significantly increased LA-induced calcium responses in mouse taste bud cells from fungiform papillae from *Adipor1*^+/+^ males; (**C**) application of adiponectin (t(89.11) = 1.988, *p* = 0.0499) and AdipoRon (t(87.25) = 2.938, *p* = 0.0042) was significantly increased LA-induced calcium responses in mouse taste bud cells from circumvallate papillae from *Adipor1*^+/+^ females; and (**D**) application of adiponectin (t(77.39) = 1.991, *p* = 0.05) and AdipoRon (t(74.92) = 2.312, *p* = 0.0235) was significantly increased LA-induced calcium responses in mouse taste bud cells from fungiform papillae from *Adipor1*^+/+^ females. Application of adiponectin and AdipoRon did not change LA-induced calcium responses in taste bud cells isolated from *Adipor1*^−/−^ mice (**E**–**H**, *p* > 0.05 for all tests, *n* = 28−100 cells from at least three animals for each group). Data are presented as mean ± SEM. Unpaired, two-tailed Student’s *t*-test (with Welch’s correction if the populations’ standard deviations are not equal) was used to determine statistical significance between the adiponectin- or AdipoRon-treated group and the LA-alone control. Asterisks correspond to significant differences between the adiponectin- or AdipoRon-treated group and the LA-alone control, * *p* < 0.05, ** *p* < 0.01.

Taste bud cells are known to highly express functional adiponectin receptors [11]. AdipoR1 and AdipoR2 are major receptors for the biological effects of adiponectin and are activated through the AMPK and PPARα pathways, respectively. Furthermore, AMPK is required to enhance AdipoRon’s role in promoting fatty acid-induced calcium responses. Thus, the enhancement effect of AdipoRon on fatty acid-induced responses is likely to be mediated by AdipoR1, as was the case for the HuFF cells [12]. To test this possibility, we performed calcium imaging to measure the cellular responses to fatty acid in isolated taste bud cells from *Adipor1*^−/−^ mice and *Adipor1*^+/+^ controls. LA-induced calcium responses in both circumvallate and fungiform taste bud cells from *Adipor1*^+/+^ mice were increased by the application of adiponectin and AdipoRon (Figure 4A–D), but not in taste bud cells from *Adipor1*^−/−^ mice (Figure 4E–H).

### 3.5. No Significant Effect of AdipoRon on Fat Taste Behavior in Mice

**Figure 5:**
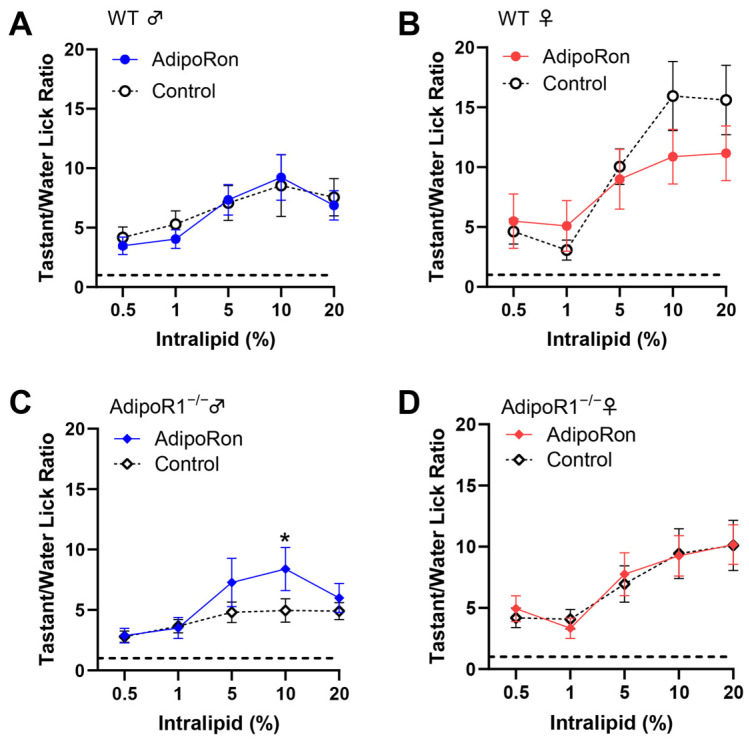
The effect of 10 μM AdipoRon on mouse behavioral taste responses to intralipid. (**A**) No significant difference in taste behavioral responses to intralipid was found in *Adipor1*^+/+^ males with or without treatment with AdipoRon (F(1, 70) = 0.1285, *p* = 0.7211); (**B**) no significant difference in taste behavioral responses to intralipid was found in *Adipor1*^+/+^ females with or without treatment with AdipoRon (F(1, 70) = 1.256, *p* = 0.2663); (**C**) an unexpected increased behavioral response to 10% intralipid (adjusted *p* = 0.0333) was found in *Adipor1*^−/−^ males treated with AdipoRon (F(1, 70) = 3.818, *p* = 0.0547); (**D**) no significant difference in taste behavioral responses to intralipid was found in *Adipor1*^−/−^ females with or without treatment with AdipoRon (F(1, 70) = 0.0212, *p* = 0.8847). The dashed lines in the graphs indicate that the tastant/water ratio is 1. Data are presented as mean ± SEM. Data between the AdipoRon-treated and control groups and intralipid concentrations were compared using two-way ordinary ANOVA with Tukey’s test for multiple comparisons. * *p* < 0.05.

Next, we performed brief-access taste tests to determine whether AdipoRon enhancement of fatty acid responses in mouse taste bud cells correlated with any differences in taste behavioral responses to fat. Since AdipoRon is an orally active agonist of adiponectin receptors, we tested AdipoRon’s ability to alter fat taste behavioral responsiveness in mice by adding it directly to the test solutions rather than injecting it. However, no significant effect in animals’ taste behavioral responses to intralipid was found in WT and *Adipor1*^−/−^ mice with (day 3 and 4) or without (day 2 and 5) the administration of 10 μM AdipoRon (Figure 5A–D). Unexpectedly, we did observe an increase in the lick ratio for a single intralipid concentration in *Adipor1*^−/−^ males treated with AdipoRon (Figure 5C).

### 3.6. Reduction of Taste Behavioral Responsiveness to Intralipid in Adipor1^−/−^ Mice

**Figure 6:**
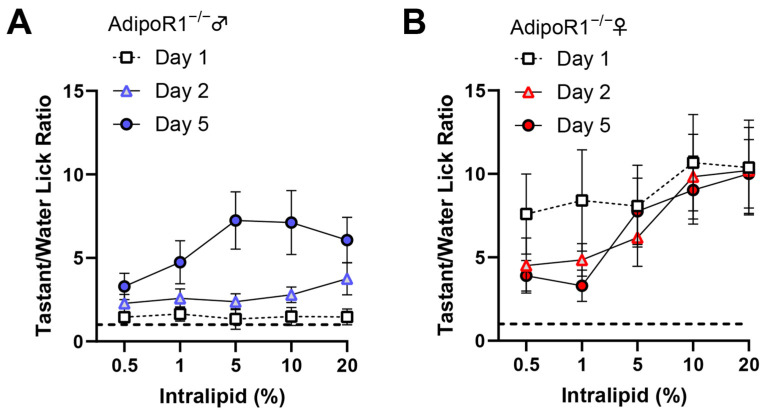
The results from brief-access taste testing of *Adipor1*^−/−^ mice differed following exposure to intralipid. (**A**) A significant difference in taste behavioral responses to intralipid was found in *Adipor1*^−/−^ male mice on different days (F(2, 105) = 25.14, *p* < 0.0001), *Adipor1*^−/−^ males failed to show concentration-dependent licking to intralipid on the first two days of testing; (**B**) no significant difference in taste behavioral responses to intralipid was found in *Adipor1*^−/−^ female mice on different days (F(2, 105) = 1.524, *p* = 0.2226). The dashed lines in the graphs indicate that the tastant/water ratio is 1. Data are presented as mean ± SEM (*n* = 8 for each group). Two-way ordinary ANOVA was used to determine statistical significance between different days and intralipid concentrations.

**Figure 7:**
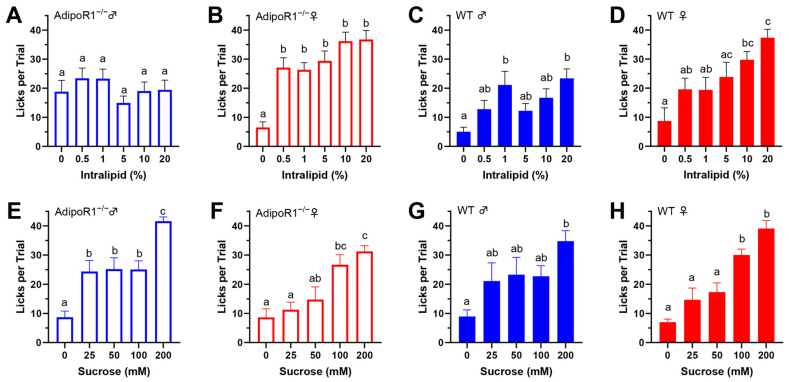
The mean number of licks per trial during brief-access tests comparing tastants and water. (**A**) There was no significant difference in linking responses to all concentrations of intralipid and pure water in *Adipor1*^−/−^ males (F(5, 42) = 0.9413, *p* = 0.4644); (**B**) *Adipor1*^−/−^ females did not show any difference in linking responses to all concentrations of intralipid but could taste the difference between water and intralipid (F(5, 42) = 14.13, *p* < 0.0001); (**C**) *Adipor1*^+/+^ males performed significantly more licks with intralipid than water in a dose-dependent manner (F(5, 42) = 4.441, *p* = 0.0025); (**D**) *Adipor1*^+/+^ females performed significantly more licks with intralipid than water in a dose-dependent manner (F(5, 42) = 6.036, *p* = 0.0003). All the animals performed significantly more licks with sucrose than with water in a dose-dependent manner, including (**E**) *Adipor1*^−/−^ males (F(4, 35) = 15.13, *p* < 0.0001); (**F**) *Adipor1*^−/−^ females (F(4, 35) = 9.901, *p* < 0.0001); (**G**) *Adipor1*^+/+^ males (F(4, 30) = 4.030, *p* = 0.0099); and (**H**) *Adipor1*^+/+^ females (F(4, 35) = 20.94, *p* < 0.0001). To better represent the fundamental differences in taste behavioral responses between water and tastant, trials were included when the number of water licks began to drop dramatically. Data are presented as mean ± SEM (*n* = 8 for each group). One-way ordinary ANOVA with Tukey’s test for multiple comparisons was used to determine statistical significance among different tastant concentrations. The letters above the bars indicate statistically significant groups within each cell type.

To reduce possible fat experience-induced differences in taste behavior that may affect the determination of AdipoRon’s effects on fat taste, we averaged the data from the day before (day 2) and the day after (day 5) to compare with the AdipoRon treatment days (day 3 and 4). The unexpected discovery in *Adipor1*^−/−^ males (Figure 5C) led us to compare each day’s data in the brief-access taste test more closely, especially for the results from mice lacking *Adipor1*. Interestingly, naïve *Adipor1*^−/−^ animals were indifferent to all concentrations of intralipid, and the fat taste impairment in *Adipor1*^−/−^ mice did not seem to be permanent; they were able to detect intralipid after being exposed to this tastant for one or two days (Figure 6). Although both *Adipor1*^−/−^ males and females lost the ability to taste different concentrations of intralipid on the first day, they responded differently to the loss of typical fat taste. *Adipor1*^−/−^ males showed very similar licking responses to all concentrations of intralipid and pure water (Figure 7A), whereas *Adipor1*^−/−^ females did not show any concentration-dependent licking to intralipid but could taste the difference between water and intralipid, even at its lowest concentration (Figure 7B).

In contrast, the WT animals performed significantly more licks with intralipid than water in a dose-dependent manner (Figure 7C,D). Additionally, when *Adipor1*^−/−^ mice were initially exposed to sucrose, they could taste the difference between the tastant and water (Figure 7E,F). Our brief-access test data suggest that knocking out AdipoR1 may have a profound effect on the ability of mice to detect fatty acids.

## 4. Discussion

The main findings from this study support the role of adiponectin signaling in peripheral gustatory fat detection in mice. Adiponectin/AdipoRon enhances LA-induced calcium responses in isolated WT mouse taste bud cells but was not seen in taste bud cells isolated from *Adipor1*^−/−^ mice. AMPK and CD36 are required for the enhancement effect of Adiponectin/AdipoRon on LA-induced calcium responses. Although the concentration (10 μM) of AdipoRon that enhanced the cellular responses of taste bud cells to fatty acids did not seem to change fat taste behaviors in mice, we found that naïve *Adipor1*^−/−^ mice failed to taste the difference between different concentrations of intralipid.

The importance of two primary fatty acid receptors, CD36 and GPR120, in the taste perception of dietary lipids is well established [26,27,28,29,30]. Both CD36 and GPR120 mediate calcium responses to fatty acids in mammalian taste bud cells [28,31], and both have been identified as critical in their contributions to fat taste [32]. Studies have shown that CD36 in other tissues can be regulated by hormones, such as GLP-2 [33], insulin [34], and adiponectin [35], to stimulate lipid uptake, while GPR120 is likely to be involved in the lipid-induced release of peptide hormones, such as GLP-1 [36], cholecystokinin (CCK) [37], and pancreatic polypeptide [38].

Given the differences in their relationships with peptide hormones, we hypothesized that the enhancement role of adiponectin/AdipoRon on fatty acid responses in taste cells is mediated by CD36. Indeed, our previous study provided evidence that the CD36 pathway, independent of the GPR120 pathway, is functionally responsible for AdipoRon’s effect on the enhancement of fatty acid-induced calcium responses [12]. Our results illustrated the inability of AdipoRon to enhance fatty acid-induced calcium responses in HuFF cells when CD36 function is pharmacologically blocked with SSO. In contrast, AdipoRon was still able to increase fatty acid-induced calcium responses in HuFF cells, blocking GPR120 function via AH-7614, and AdipoRon was unable to affect calcium responses induced by the GPR120 agonist GW9508 [12]. Studies in skeletal muscle cells and cardiomyocytes have suggested that CD36 is regulated by adiponectin signaling in two distinct ways: (1) by increasing the expression of CD36 [39]; and (2) by enhancing the translocation of CD36 from the intracellular region to the plasma membrane [35]. AdipoRon/adiponectin’s enhancement of fatty acid-induced calcium responses in taste cells occurs within several minutes. Therefore, the time course of changes in CD36 gene expression may not fit with the acute effect of AdipoRon/adiponectin in taste cells. Results from our previous research also indicated that CD36 subcellular translocation, rather than CD36 transcription, is responsible for the enhancement effect of AdipoRon on fatty acid responses in HuFF cells [12]. We would expect to find similar mechanisms in mouse taste systems; however, in the present study, we focused only on the involvement of CD36 in the effect of AdipoRon/adiponectin on fatty acid-induced calcium responses in mouse taste bud cells. As expected, pharmacological blocking of CD36 function by SSO pretreatment eliminated the enhancement role of adiponectin/AdipoRon on fatty acid-induced calcium responses in taste bud cells isolated from wild-type mice, indicating the involvement of CD36 in adiponectin signaling on fat sensing in mice. Future studies are needed to address whether GPR120 signaling interacts with adiponectin signaling to influence fat taste modulation and whether adiponectin/AdipoRon stimulates CD36 translocation in mouse taste bud cells.

We also demonstrated that pharmacological inhibition of the AMPK pathway via Compound C attenuated AdipoRon’s enhancement effect on fatty acid responses in isolated wild-type mouse taste bud cells. AMPK is essential in regulating energy homeostasis, which mediates adiponectin-stimulated fatty acid oxidation in skeletal muscle [1]. Adiponectin has been reported to increase AMPK activity in the hypothalamus to stimulate food intake in mice [6]. Moreover, the presence of CD36 is required for AMPK-mediated stimulation of long-chain fatty acid uptake in cardiomyocytes [40], and AMPK promotes long-chain fatty acid uptake in intestinal epithelial cells by simultaneously regulating CD36 protein abundance and subcellular translocation [41]. In addition, adiponectin-stimulated long-chain fatty acid uptake in adult cardiomyocytes was significantly attenuated by inhibition of the AMPK pathway with Compound C [35]. These studies indicate that the AMPK-CD36 pathway is involved in adiponectin signaling. AdipoRon has been shown to increase the phosphorylation of AMPKα (Thr172), and AdipoRon-enhanced CD36 cell surface translocation was effectively eliminated by Compound C in HuFF cells [12]. Since both AMPK and CD36 are required for the enhancement role of adiponectin signaling on fatty acid responses in mouse taste bud cells, a similar mechanism may function in the mouse taste system, in which AdipoRon/adiponectin-stimulated activation of AMPK could lead to the CD36 translocation from the intracellular to the plasma membrane, thereby increasing fat taste responses.

Adiponectin undergoes post-translational modifications to form trimers, hexamers, high molecular weight (HMW) multimers, and super-HMW multimers (specific to saliva samples) [42,43]. At least three receptors, AdipoR1, AdipoR2, and T-cadherin, have been shown to mediate the pleiotropic actions of adiponectin [21,44]. As one of the best-known and most abundant hormones in the plasma, the importance of adiponectin is well-established in many tissues and organs [45]. Adiponectin has received much attention for its role in various metabolic processes (including uptake and oxidation of lipids and carbohydrates) by stimulating the activity of AMPK and PPARα through the AdiopoR1 and AdipoR2 receptors, respectively [23]. Decreased adiponectin levels have been found in patients with chronic diseases, such as obesity [46], type 2 diabetes [47], and hypertension [48]. Interestingly, the loss of taste has also been demonstrated in such disorders [49,50].

**Figure 8:**
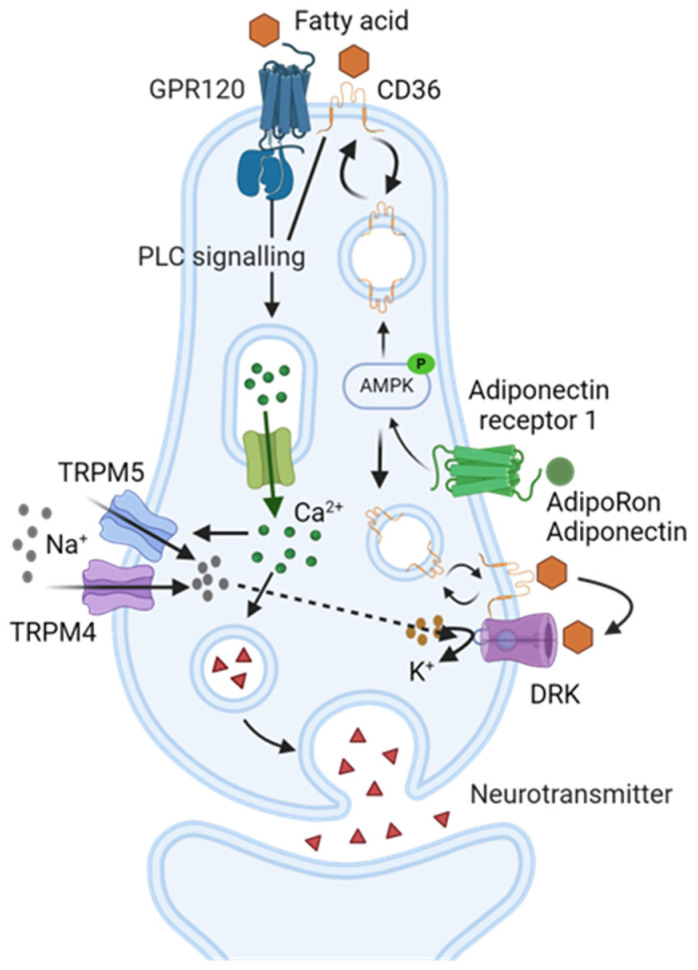
Proposed mechanism of adiponectin signaling on fat sensing in taste cells. Adiponectin (or AdipoRon) binds to adiponectin receptor 1 (AdipoR1), which stimulates the activation of 5′ adenosine monophosphate-activated protein kinase (AMPK). In response to stimulation with adiponectin (or AdipoRon), cluster of differentiation 36 (CD36) translocates from intracellular storage depots to the cell membrane to increase fatty acid taste signaling. Fatty acids bind to their G protein-coupled receptors on the cell membrane, release G proteins, stimulate phospholipase C-beta 2 (PLCβ2), generate second messengers, elicit an increase in intracellular calcium, activate transient receptor potential channel M4 and/or M5 (TRPM4/TRPM5), and induce cell depolarization. CD36 and fatty acids may interact directly with PLCβ signaling, or CD36 may facilitate fatty acid binding with the receptors (GPR120) to enhance the taste cell activation or block fatty acid-sensitive delayed rectifying K+ (DRK) channels to prolong depolarization of the cell. Created with BioRender.com.

Adiponectin receptors are highly expressed in mammalian taste buds [11]. Therefore, it is plausible that adiponectin signaling could play a critical role in taste regulation like other hormones, such as leptin, GLP-1, and ghrelin. However, little is understood about the mechanisms of adiponectin signaling modulation in the taste system due to the complex nature of adiponectin signaling. In the present study, we targeted AdipoR1 to study the role of adiponectin signaling in fat taste for several reasons, as mentioned in the introduction: (1) T-cadherin is the receptor for the HMW form of adiponectin [44], and AdipoRon is an agonist of AdipoR1 and AdipoR2 [51], therefore T-cadherin is unlikely to be a contributor to the effects of AdipoRon on fatty acid responses; (2) the EC_50_ value of AdipoRon’s enhancement of LA-induced calcium responses (1.67 µM) [12] is close to the Kd values reported for AdipoRon binding to AdipoR1 (1.8 µM) and AdipoR2 (3.1 µM) [51], suggesting a potential role of these receptors in the effects of AdipoRon on fat taste responses; (3) however, AdipoR1 and T-cadherin immunolocalize to mouse circumvallate taste buds, while AdipoR2 immunolocalize to the surrounding tissues, but not taste buds [11]; (4) adiponectin receptors mediate distinct downstream signaling pathways: AdipoR1 appears to be more tightly associated with the activation of the AMPK pathway, whereas AdipoR2 is more closely involved in the activation of the PPARα pathway [23,51]; (5) the effect of AdipoRon on LA-induced calcium responses in HuFF cells requires the activation of AMPK [12], and adiponectin failed to active AMPK in hepatocytes of *Adipor1*^−/−^ mice [23]; (6) last, but not least, a similar mechanism of adiponectin stimulating fatty acid uptake by regulating CD36 via the activation of AdipoR1 and AMPK has been demonstrated in other tissues. Combined with previously published data [12], our experiments show that adiponectin/AdipoRon enhances fatty acid-induced calcium responses in taste cells via CD36 and the increases in CD36 translocation induced by adiponectin/AdipoRon that occur through AdipoR1-AMPK signaling pathway [35]. Therefore, we postulate a novel pathway by which adiponectin signaling modulates fat taste responsiveness in mammalian taste cells (Figure 8). Although our results indicated that AdipoR1 is the contributor to the role of AdipoRon/adiponectin in fatty acid responses in taste cells, we cannot exclude other receptors, especially T-cadherin, which may also play an essential role in mammalian taste sensation. Additionally, HMW multimers have been suggested to be the primary active form mediating the multiple metabolic actions of adiponectin in peripheral tissues. Because saliva specifically contains a super high molecular weight form of adiponectin [43], and T-cadherin is highly expressed in taste bud cells [11], further studies are needed to fully understand the exact role of adiponectin signaling in the peripheral gustatory system.

Our results showed that adiponectin/AdipoRon enhances fatty acid-induced calcium responses through AdipoR1–AMPK–CD36 signaling in taste cells, and we hypothesize that AdipoRon plays a physiological role in the modulation of fat taste. We used a brief-access taste testing assay to test the effects of AdipoRon on behavioral responses to intralipid in WT and *Adipor1*^−/−^ mice. However, in our experiments, the AdipoRon concentration (10 μM) that enhanced the cellular responses of taste bud cells to fatty acids did not appear to alter the intralipid/water ratio in brief-access testing in mice. It is worth noting that the cell-based experiments were performed in vitro in the absence of endogenous adiponectin. In contrast, the behavioral studies were conducted in animals rich in salivary and circulating adiponectin. It has been reported that adiponectin knockout mice and WT mice have equivalent taste behavior responses to different taste stimuli, including intralipid; and the salivary gland-specific adiponectin rescue, but not the global adiponectin rescue, in adiponectin KO mice significantly increases behavioral taste responses to intralipid [11]. Therefore, our results are not particularly surprising, because AdipoRon’s effect is likely masked by endogenous adiponectin, especially salivary adiponectin. Thus, without affecting endogenous adiponectin, adiponectin KO mice should be an effective animal model to verify the in vivo effect of AdipoRon on fat taste. Moreover, it is possible that the concentrations we used were insufficient to elicit differences in taste behavior or that the drug delivery method we applied was inappropriate to induce behavioral changes in taste. Higher concentrations of AdipoRon or other drug delivery approaches are needed to elucidate the functional role of AdipoRon in regulating fat taste.

No significant differences were found in the taste behavioral assay in WT animals treated with AdipoRon. If anything, there was a trend toward AdipoRon in female mice altering licking responsiveness in brief-access taste tests. A recent study reported that male, but not female, diabetic mice were resistant to globular adiponectin (gAd) treatment and that gAd-treated female, but not male, mice displayed higher AMPK activity and CD36 protein levels compared to controls [52]. Adiponectin levels are higher in females than males [53,54]. Recently, it was shown that differing adiponectin concentrations may account for sex differences in human olfactory sensitivity [55]. Data from our lab showed that females had higher fat taste sensitivity and could detect lower concentrations of fatty acids [24]. It is still unclear if and how adiponectin signaling may influence fat taste detection ability in a sex-dependent manner. Further studies are needed to explore whether the sex differences in fat taste sensitivity and adiponectin concentrations are related.

Although our experiments could not provide direct evidence that AdipoRon affects fat taste behavior, the data from brief-access testing showed that fat-naïve *Adipor1*^−/−^ animals were indifferent to all concentrations of intralipid, suggesting adiponectin signaling has a profound effect on the ability of mice to detect fatty acids. Unlike *Adipor1*^−/−^ females, who performed more licks per trial of water with intralipid than pure water, *Adipor1*^−/−^ males did not show any significant difference in licks comparing intralipid and water, indicating a sex-dependent role of adiponectin signaling on fat taste behavior or a sex-dependent response strategy in the face of fat taste loss. Knocking out *Adipor1* may alter critical downstream cellular signaling in different ways between males and females, and there may be compensatory mechanisms that can supplement the lack of adiponectin signaling in females. Our cell-based experiments showed that adiponectin signaling enhances the cellular responses to fatty acids in taste cells. We expected that *Adipor1* deficiency would reduce behavioral responses to fat stimuli in mice; however, the completely abolished taste behavioral responses to intralipid in *Adipor1*^−/−^ animals in our experiments were unexpected, since these mice should contain intact fat taste signaling components. Reduction of CD36 expression on the tongue has been shown to attenuate linoleic acid preference in obesity-prone and obesity-resistant rats [56].

Similarly, CD36 knockout mice failed to prefer fat emulsion in both short- and long-term two-bottle tests [30], and fat preference was fully abolished not only in CD36 knockout mice but also in heterozygous CD36^+/−^ animals (two-fold lower CD36 protein level in circumvallate papillae) [29]. Recently, it was reported that CD36 protein levels were significantly reduced (3.7-fold) in the retinas of *Adipor1*-deficient mice [57]. Our behavioral studies demonstrated that fat taste loss was found only in *Adipor1* knockout mice but not in WT animals, and the *Adipor1* knockout animals were indifferent to intralipid but not to sucrose. AdipoRon/adiponectin activates AMPK through AdipoR1 and increases the translocation of CD36 from intracellular sites to the cell membrane, thereby selectively enhancing cellular responses to fatty acids. Therefore, if true, lower CD36 protein levels in taste bud cells, combined with the lack of adiponectin signaling to stimulate CD36 translocation to maintain certain cell surface expression levels, may contribute to the absence of fat behavioral responses in *Adipor1*-deficient mice.

After being exposed to the intralipid for one (females) or two (males) days in the brief-access test, *Adipor1*^−/−^ mice could detect the different concentrations of intralipid. CD36 gene and protein levels in mouse circumvallate papillae were sensitive to the dietary lipid content and affected by dietary intake during a fasting/re-feeding sequence [29]. Therefore, changes in CD36 levels induced by lipid exposure may contribute to recovery of the ability to taste fatty acids. In addition, we cannot exclude the possibility of the post-oral nutritive effects attributed to the taste recovery. Experience-induced recovery of fat preference has been found in taste-deficient animals with genetic knockout of gustatory fat taste signaling components such as CD36, CALHM1, TRPM5, and P2X2/P2X3. These animals could learn to use residual oral fat sensory cues based on post-oral reinforcing actions to display strong preferences for and high intakes of intralipid [58,59,60,61]. Further studies are needed to understand better the role of adiponectin signaling in fat taste and to explore the links among adiponectin signaling, fat taste, dietary fat intake, and obesity.

## 5. Conclusions

In this report, our results indicate that AdipoRon/adiponectin increased taste responses to fatty acids in taste cells by activating AdipoR1 and AMPK. Although the concentration of AdipoRon (10 μM) that enhanced the cellular responses of taste bud cells to fatty acids did not appear to alter the animals’ behavior toward fatty acids, brief-access taste testing in *Adipor1*-deficient mice showed that naïve *Adipor1*-deficient male mice failed to taste the intralipid solutions. In contrast, naïve *Adipor1*-deficient female animals could detect the difference between intralipid and water but were indifferent to increasing concentrations of intralipid. These findings suggest that adiponectin signaling has a profound, possibly sex-dependent, function in regulating fat taste.

## Data Availability

All relevant data are within the manuscript.

## Supplementary Materials

The following supporting information can be downloaded at: https://www.mdpi.com/article/10.3390/nu16213704/s1, Figure S1: Body weight and total body composition in *Adipor1*^+/+^ and *Adipor1*^−/−^ mice.
