# Supplementary material for: Adiponectin Signaling Modulates Fat Taste Responsiveness in Mice
Source: Nutrients. 2024 Oct 30;16(21):3704. doi: 10.3390/nu16213704 (PMC11547430; doi:10.3390/nu16213704)
Supplement: Supplementary file 1 [file nutrients-16-03704-s001.zip › nutrients-3251355-supplementary.pdf]

nutrients

MDPI

Article

Adiponectin Signaling Modulates Fat Taste Responsiveness in Mice

Fangjun Lin [1,2], Emeline Masterson [1,2] and Timothy A. Gilbertson [2,*]

Supplementary Information

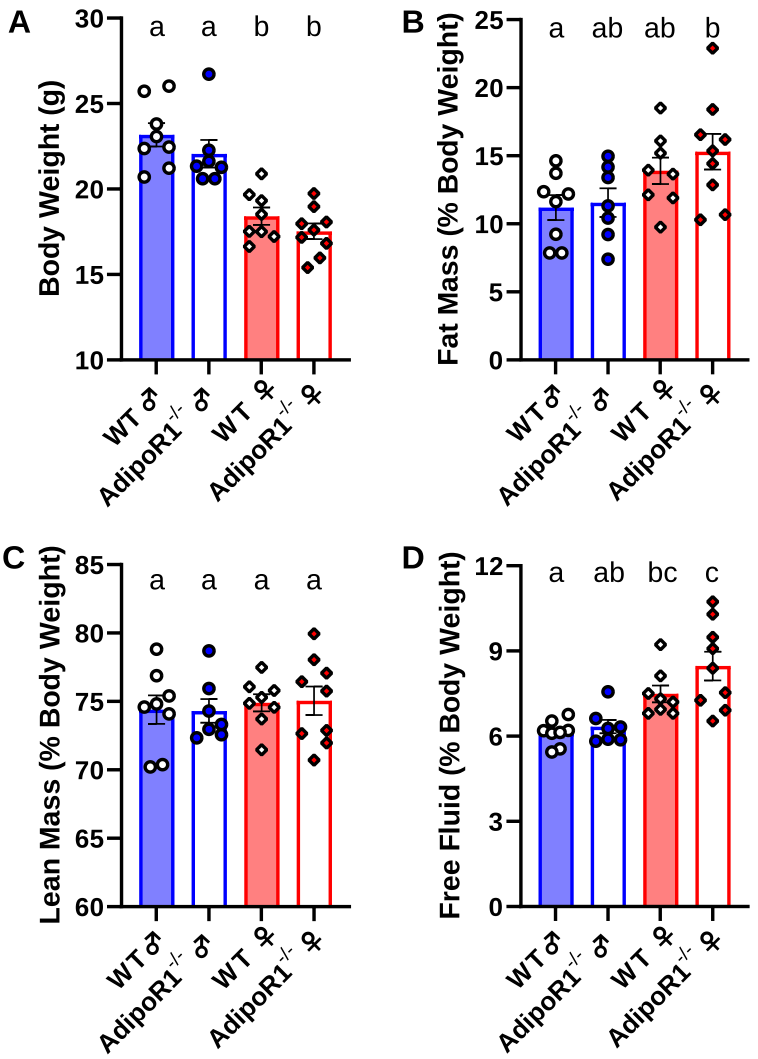

Figure S1. Body weight and total body composition in AdipoR1+/+ and AdipoR1-/- mice. (A) There was no significant genotype effect on the body weight of animals at about six weeks of age (F (1, 28) = 2.655, P=0.1144), but there was a sex difference (F (1, 28) = 57.76, P<0.0001); (B) There was no significant genotype effect on the percentage of fat mass in animals (F (1, 28) = 0.646, P=0.4281), but there was a sex difference (F (1, 28) = 8.633, P=0.0065); (C) No significant difference was found in the percentage of lean mass among the animals (genotype effect: F (1, 28) = 0.0008), P=0.9774, and gender effect: F (1, 28) = 0.4542, P=0.5059); (D) There was no significant genotype effect on the percentage of free fluid in animals (F (1, 28) = 3.014, P=0.0936), but there was a sex difference (F (1, 28) = 25.53 , P<0.0001). Data are presented as mean ± SEM (n = 7–9 mice for each group). Two-way ordinary ANOVA with Tukey's test for multiple comparisons was used to determine statistical significance. Letters above bars indicate statistical grouping.
